# Detrended Fluctuation Analysis and Adaptive Fractal Analysis of Stride Time Data in Parkinson's Disease: Stitching Together Short Gait Trials

**DOI:** 10.1371/journal.pone.0085787

**Published:** 2014-01-23

**Authors:** Marietta Kirchner, Patric Schubert, Magnus Liebherr, Christian T. Haas

**Affiliations:** Faculty of Health and Social Sciences, Hochschule Fresenius, University of Applied Sciences, Idstein, Germany; National Institute of Genomic Medicine, Mexico

## Abstract

Variability indicates motor control disturbances and is suitable to identify gait pathologies. It can be quantified by linear parameters (amplitude estimators) and more sophisticated nonlinear methods (structural information). Detrended Fluctuation Analysis (DFA) is one method to measure structural information, e.g., from stride time series. Recently, an improved method, Adaptive Fractal Analysis (AFA), has been proposed. This method has not been applied to gait data before. Fractal scaling methods (FS) require long stride-to-stride data to obtain valid results. However, in clinical studies, it is not usual to measure a large number of strides (e.g., 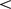 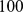 strides). Amongst others, clinical gait analysis is limited due to short walkways, thus, FS seem to be inapplicable. The purpose of the present study was to evaluate FS under clinical conditions. Stride time data of five self-paced walking trials (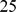 strides each) of subjects with PD and a healthy control group (CG) was measured. To generate longer time series, stride time sequences were stitched together. The coefficient of variation (CV), fractal scaling exponents 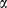 (DFA) and 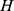 (AFA) were calculated. Two surrogate tests were performed: A) the whole time series was randomly shuffled; B) the single trials were randomly shuffled separately and afterwards stitched together. CV did not discriminate between PD and CG. However, significant differences between PD and CG were found concerning 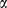 and 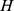. Surrogate version B yielded a higher mean squared error and empirical quantiles than version A. Hence, we conclude that the stitching procedure creates an artificial structure resulting in an overestimation of true 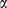. The method of stitching together sections of gait seems to be appropriate in order to distinguish between PD and CG with FS. It provides an approach to integrate FS as standard in clinical gait analysis and to overcome limitations such as short walkways.

## Introduction

The ability to walk is a key component of mobility and is highly related to quality of life. Its assessment enables to get insight into system behaviour and gait disorders. In Parkinson's disease (PD), impaired gait is well documented with patients showing various gait abnormalities [1]–[4]. From a biomechanical point of view, gait disorders in PD can be characterised by spatiotemporal regulation difficulty e.g., shortened stride length and reduced stride velocity [5], [6]. Quantification of within-subject stride-to-stride changes have proven to be promising in terms of characterising gait disturbances in PD [1]. Previous studies have found an increased stride-to-stride variability in patients with PD compared to controls with the tendency of increasing variability with disease severity [7], [8]. In general, stride time variability has been shown to be affected by disease and ageing [8]–[10]. Quantification of stride-to-stride variability requires to measure a huge number of strides - the exact number is not known - than needed when analysing average stride characteristics [11], [12].

Concerning the analysis of variability, a new perspective has been established in the last years. Besides the quantification of the amount of variability (e.g., coefficient of variation), the structure has been quantified in order to capture the dynamical properties of the system (for review, see [13], [14]). It provides additional information and has been proven sensitive in detecting subtle changes of the system. For instance, [15] could distinguish elderly with more severe gait disorders from healthy age-matched controls by examining gait variability. However, among the subjects with more severe gait disorders, only the structural parameter was able to divide this group into fallers and non-fallers. The combined application of linear and nonlinear tools yields a complementary characterisation of gait variability and how it changes with age and disease [16]. In order to quantify the structure of stride-to-stride variability, Detrended Fluctuation Analysis (DFA) was previously applied [11], [15], [17], [18] and especially with respect to stride time variability in PD [1], [2], [19]. DFA was introduced by [20] as a method to quantify the fractal dynamics or self-similarity of a time series. The method outputs the scaling exponent 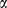 which can be interpreted in terms of correlations [21], [22]. That is, 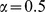 is characteristic of an uncorrelated signal and 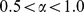 of a persistent signal (positive correlation). In healthy subjects walking under self-paced condition, a fractal scaling index of around 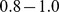 was observed [1], [10], [23] and higher indices resulted when walking slower or faster than self paced [23]. Values closer to 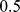 reflect a deviation from a healthy state and more random dynamics [9], [10], [24], [25]. It could be shown that PD patients have a DFA scaling exponent close to 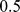 which indicates that stride-to-stride fluctuations are more random and that the long-range scaling behaviour is reduced [1], [19]. A simple explanation is that gait of PD patients looses its automatism and fluidity with a break down of memory of the locomotor control system [1]. [19] showed that the 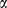-value decreases from control group to early PD to later PD patients which underlines the decrease of long-range scaling with disease severity. DFA is just one example to obtain structural information from time series data. Recently, a new method has been proposed, adaptive fractal analysis (AFA) [26]–[28], which is similar but has a number of advantages over DFA. We would like to point out two of them. First, the most important difference is, that AFA identifies a global smooth trend of the data by combining segments of overlapping windows, whereas in DFA the result of the linear fitting resembles a discontinuous signal with abrupt jumps. Therefore, AFA is not restricted on the signal being stationary. Second, AFA presents a more robust method concerning short time series compared to DFA [26], [27]. While DFA is one of the most applied method with respect to stride interval time series, AFA has not been applied for this purpose before. It may be a valuable procedure to compare the results of DFA with AFA fractal scaling outcomes in this context.

The accuracy of the estimation of fractal exponents is reduced related to the length of the time series [29]–[31]. [30] propose that one needs series of at least 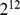 data points to get reliable results. However, [32] showed that the loss of accuracy of the estimation in short time series (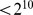) is not as dramatic as expected. In clinical gait analysis a few number of strides are typically recorded (e.g., 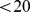 strides [33], [34]), which can be due to a short walkway (e.g., GAITRite system [35], [36]). [36] review that studies differ with respect to the measured number of strides with a reduced reliability when only a few number of strides are analysed. Thus, typical clinical studies of gait are not suitable to the premise of needing long time series for DFA. However, [37] analysed the effects of nonstationarity on DFA, i.e., stitching together segments of data obtained from discontinuous experimental recordings. They found that positively correlated signals with 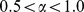, which can be expected for stride time variability [11], are not affected by the cutting procedure. [1] applied this procedure on experimental data - he analysed gait of subjects walking on a circuit and cut out the turn - resulting in longer time series which only include the straight walking distances. Following these works, the aim of this study is to evaluate the quantification of stride time variability by use of DFA, AFA and the coefficient of variation in patients with Parkinson's disease compared to a healthy control group based on stitching together consecutive walking trials in order to generate longer time series.

## Materials and Methods

In this study, 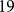 patients with Parkinson's disease (PD) (age: 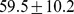; UPDRS: 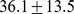; Hoehn and Yahr stages I and II) and a control group (CG) of 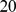 healthy younger subjects (age: 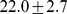 years) participated voluntarily and gave written informed consent to the experimental procedure. Gait analysis of PD was part of a larger study protocol which was approved by the ethics committee of the Hochschule Fresenius, University of Applied Sciences, Idstein, Germany and complies with the scope of the declaration of Helsinki. PD patients with deep brain stimulation, further neurological diseases, orthopaedic impairments, with advanced dementia, and/or inability to walk autonomously were excluded. PD subjects were measured under regular medication (on-state). The subjects were instructed to walk at their individual self-paced velocity along a 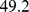 m corridor. Wireless Medilogic© foot pressure insoles were used to evaluate the heel strike time of each foot. The sample rate was set to 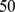 Hz. With regard to gait initiation [38], recommend to start data collection after two complete gait cycles in order to achieve steady-state walking. [39] found that a 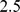 m distance is sufficient even with frail people. In the present study, the measurement was started after a 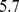 m gait initiation phase when the subjects crossed a predefined line to achieve steady-state walking. Measurement was completed 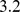 m before the end of the corridor, when the subjects crossed a second line, to exclude the gait deceleration phase. One practice walk and five trials were conducted. The heel strike times lead to a right food and a left food time series of single stride durations for each trial. There was no evidence for freezing, festination, or common concomitants associated with PD [40]. Data analysis was conducted via Matlab© 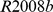.

**Figure 1:**
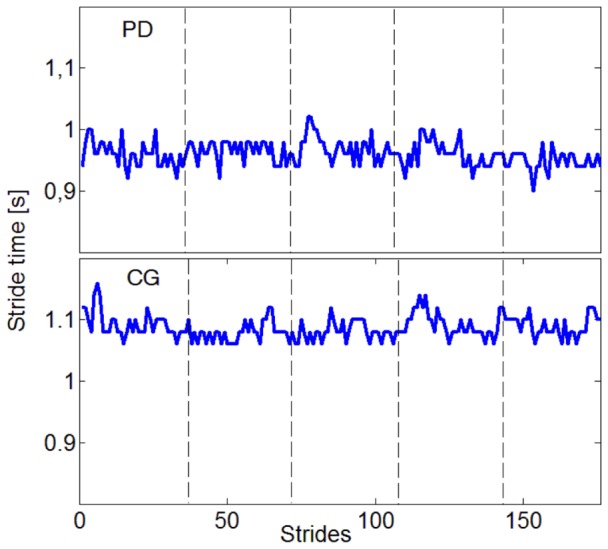
Exemplary stride-to-stride time series of a Parkinson's disease patient (PD) and a healthy subject (CG) after the stitching procedure. The vertical dashed lines represent the stitching position.

**Figure 2:**
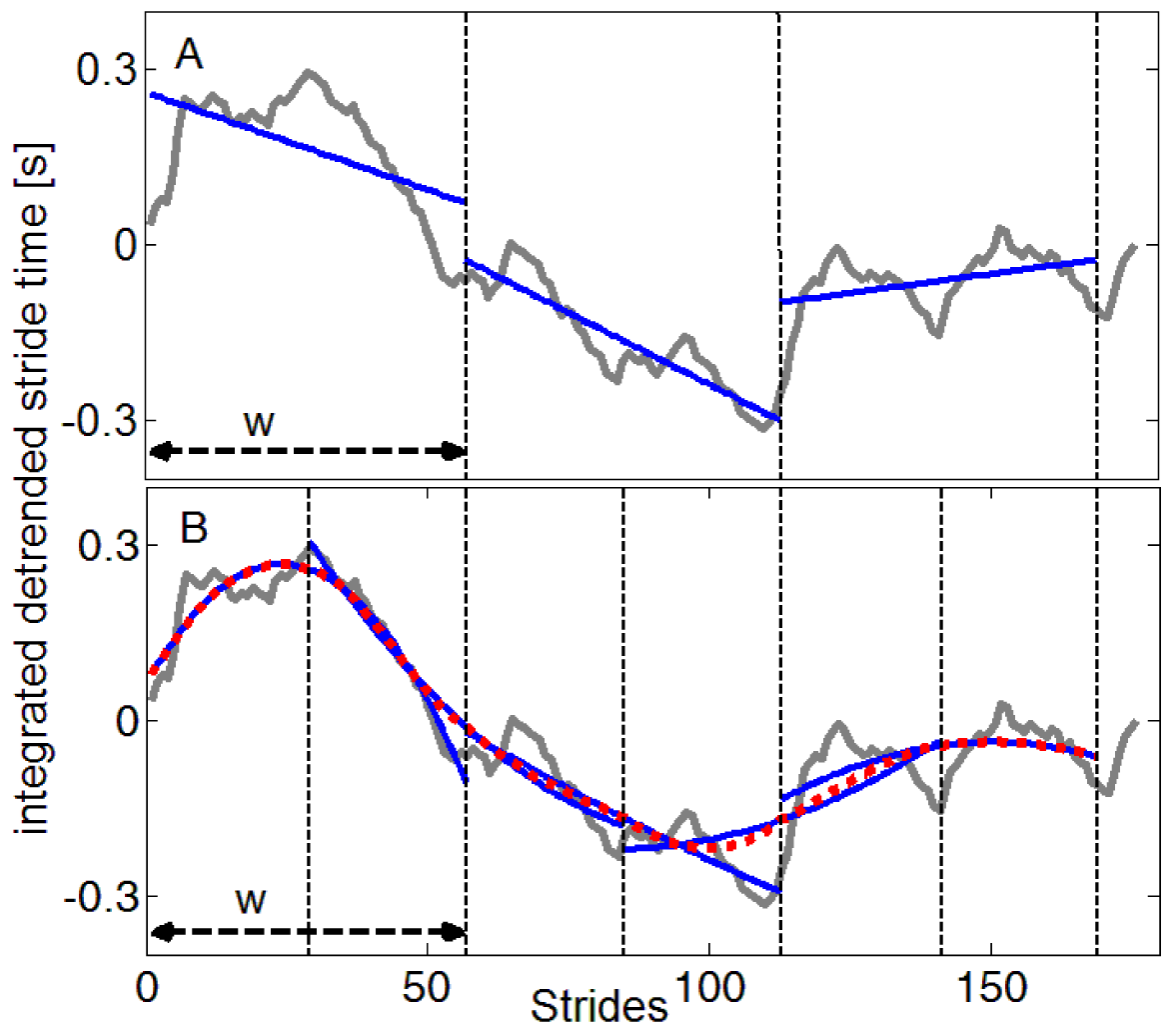
Polynomial fitting methods of DFA (A) and AFA (B). Example of an integrated detrended stride time series (grey) and a window size of 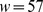. In the case of DFA, linear regression lines (blue) are computed within nonoverlapping windows resulting in a discontinuous signal. In the AFA method overlapping windows of 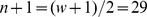 data points are used. Within each window a quadratic regression line 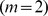 is fit to the time series (blue). Afterwards a global smooth and continuous trend (dotted red line) is calculated.

One gait trial consists of about 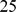 strides (data points). By stitching the five trials together, longer data series were constructed with a total number (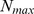) of 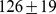 (PD) and 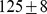 (CG) data points. The stitching procedure comprised the simple addition of consecutive trials. Suppose 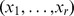 is the time series of the first trial and 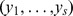 is the time series of the subsequent trial, then the stitched time series is 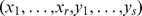 (Figure 1). To quantify the stride-to-stride variability, the coefficient of variation (CV [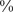]) as well as the fractal scaling exponents 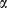 by means of DFA with linear detrending and 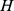 by means of AFA with quadratic polynomial fits 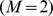 were computed (Figure 2). The linear regression is the typical procedure for DFA. In case of AFA [27], propose to use a linear or a quadratic trend, as not every variation of the signal should be captured, leaving enough residuals to analyse further. We created smooth signals for both, 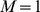 and 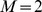 to visually define the best polynomial order. The linear trends produced inappropriate fits at the edges (region of no overlap), whereas the quadratic polynomials produced more accurate regressions.Time series were integrated prior to the application of DFA or AFA. Technical details of DFA and AFA are extensively described elsewhere (e.g., [20], [27], [28], [32]). After visual inspection of the log-log-plot, scaling exponents were determined as the slope of the linear regression line over the window sizes 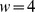 to 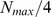 in steps of 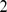 for DFA and 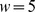 to 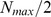 (or 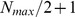 if the time series has an even number of samples) in steps of 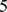 in the case of AFA. Concerning the parameter CV, the intra class correlation coefficient ICC(3,1) for each foot was calculated in order to quantify the trial-to-trial reliability.

**Figure 3:**
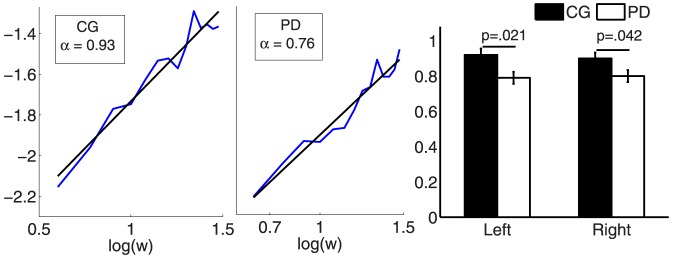
Empirical statistical bounds (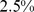 and 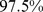 quantiles: 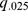, 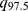) for surrogate versions A (left panel) and B (right panel). As an example, the distribution of 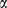 computed from surrogate series of stride time data of one subject is presented with a mean value of 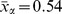 for version A and 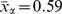 for version B. The respective mean squared errors are 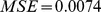 (A) and 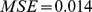 (B).

The parameters (CV, 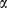, and 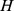), were tested for statistically significant differences between the two groups. In case of normally distributed data - proved by the Shapiro-Wilk-Test - the t-Test was applied, and otherwise, the Mann-Whitney-U-Test. The significance level was set to 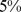. In addition, linear correlation between CV, 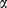, and UPDRS was determined by means of Pearson (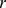) or Spearman (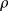) correlation coefficient. With respect to DFA and AFA, surrogate data tests were applied to test the null hypothesis (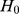) of 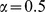 (uncorrelated series) [22], [41], independently for every subject. Thus, 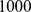 realizations were generated for each subject and the lower (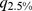) and upper (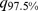) sample quantiles were computed (Figure 3). Two different versions were applied: A) the whole time series was randomly shuffled; B) the single trials were randomly shuffled separately and afterwards stitched together. Version B was applied to look for artefacts of the method of stitching together the five trials. The bias (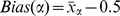) together with the mean squared error (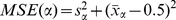) were determined to evaluate the goodness of the estimation. Correlations between 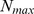 and 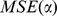 were conducted to test for significant relations between the length of the time series and the error of the estimation. This is an exploratory study where descriptive 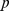-values are reported with 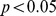 considered significant.

## Results

Results of the right (R) and left (L) stride time variability are presented in the following. Normal distribution was accepted for the data. Results are presented as mean 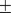 standard deviation. In order to account for several studies that have shown a close relationship between fractal scaling exponents calculated on stride times and walking speed (e.g., [11], [42]), mean velocity of the gait trials (self-paced) were calculated and found to be 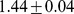 m/s for PD and 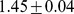 m/s for CG. 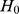 of being equal sets of walking trials could not be rejected 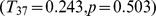. However, the five gait trials exhibited different gait velocities for both groups which was tested by use of oneway repeated-measures ANOVA (PD: 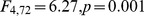; CG: 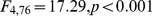) showing the tendency that gait velocity increases with trial number.

### Stride-to-stride variability

A reduced CV*_L_* sample mean was observed in PD (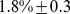) compared to CG (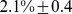) which was not significantly different (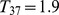, 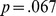). Concerning CV*_R_*, a sample mean of 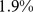 (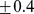) for PD and 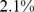 (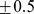) for CG was observed with no significant differences (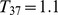, 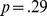). ICC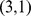 shows rather poor values in PD (L: 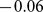, R: 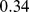) as well as in CG (L: 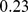, R: 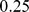). CV values of the five gait trials are shown in Table 1.

**Table 1:** Sample mean 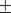 standard deviation of the coefficient of variation (CV [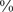]) of each gait trial.

Trial	CG		PD	
	Left	Right	Left	Right
1	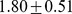	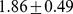	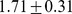	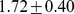
2	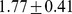	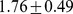	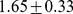	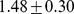
3	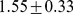	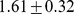	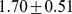	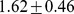
4	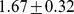	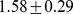	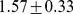	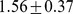
5	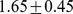	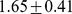	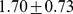	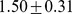

(CG = control group, PD = Parkinson's disease group).

**Figure 4:**
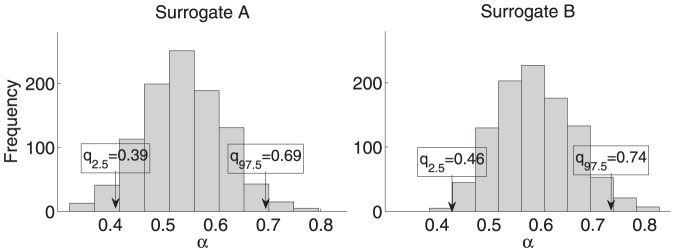
Results of the fractal scaling exponent 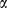, determined by DFA, of the Parkinson's disease patients (PD) and the control group (CG). Example of one subject respectively (left and middle panel). Right panel: sample mean 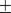 standard error of 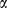. The p-value is reported concerning the statistical comparison of the two groups (CG = black, PD = weight).

Concerning the scaling exponent 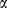, PD showed significantly lower values (R: 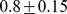, L: 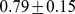) compared to CG (R: 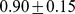, L: 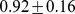) as presented in Figure 4. In accordance to the results of DFA, AFA exhibited significantly lower values for PD (R: 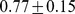, L: 
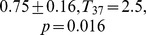
) in contrast to CG (R: 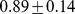, L: 
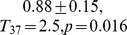
). An exemplary time series with its global smooth trend and the log-log-plot are shown in Figure 1.

Correlation between UPDRS, CV, and 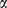 for PD and between CV and 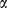 for CG are presented in Table 2 with respect to right and left stride time variability. No significant correlations were found.

**Table 2:** Results of Pearson (r) and Spearman (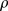) correlation coefficient of right and left stride time data concerning coefficient of variation (CV), scaling exponent 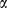 computed with DFA and UPDRS score.

Correlation	CG		PD	
	Left	Right	Left	Right
CV, DFA	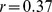	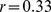	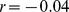	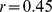
CV, UPDRS			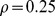	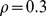
DFA, UPDRS			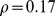	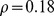

No significant (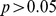) correlations were found. CG = control group, PD = Parkinson's disease patients.

### Surrogate data tests

Surrogate data tests were used to test the null hypothesis of 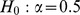 separately for every subject. With respect to surrogate version A, 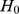 could not be rejected for 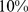 of CG and for 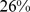 of PD concerning DFA and could not be rejected for 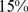 of CG and 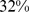 of PD in the case of AFA. Concerning surrogate version B, 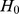 could not be rejected for 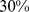 of controls and for 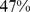 of patients with respect to DFA, and could not be rejected for 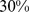 of CG and 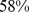 of PD. The mentioned percentages were true for both, left and right stride time data. The comparison of the two surrogate versions, A and B, yielded a higher bias or mean squared error when the single trials were shuffled separately and afterwards stitched together. That was, for 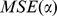 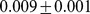 and 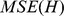 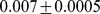 (A) versus 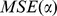 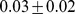 and 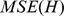 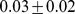 (B). For 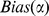 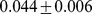 and 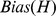 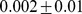 (A) versus 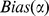 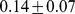 and 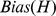 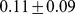 (B). In addition, version B yielded increased statistical bounds 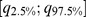 for the acceptance region of 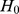: on average 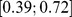 for DFA and 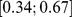 for AFA(A) versus 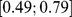 for DFA and 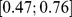 for AFA (B) (Figure 3). Concerning version A, smaller MSE values for longer time series were observed: a significant correlation between 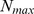 and 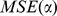 was obtained (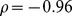, 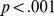). This was not the case with respect to surrogate version B (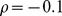, 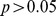).

## Discussion

Under clinical conditions, the application of fractal methods to stride time data is often difficult due to the need of long continuous recordings to attain the true value of the scaling expontent. In clinical standard diagnosis, there is often a lack of space which counteracts the evaluation of a large number of strides. Hence, in this study, we examined whether stitching together short sequences of stride time data illustrates a reasonable method to generate sufficiently long time series for the application of fractal methods. To test this procedure, a cohort of PD subjects and a healthy control group were measured. Two fractal methods, DFA and AFA, were applied to stride time series to account for differences between both subject groups. In addition, the CV was calculated as a linear and frequently used parameter of stride time variability data.

CV of stride time data in healthy adults is about 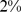 [1], [18], [43] which fits to our results. Interestingly, the data of our PD patients reached lower values which is contrary to the literature [4], [8], [44]–[46]. For instance [44], found CV values of 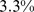 (non-fallers) to 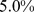 (fallers) for PD patients in on-state. Our examination of variability differs from these studies with respect to methodology. Moreover, in the present study, only subjects with low disease severity (low UPDRS score, Hoehn and Yahr stage I and II) were included. [4] report higher values for PD fallers (CV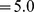) versus nonfallers (CV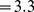) under medication. In addition, we found that CV of stride time has a low trial-to-trial reliability. Gait data exhibited very poor values of ICC. This is comparable to [47] who found that the coefficient of variation of stride time in healthy older adults (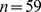) is attended by a low test-retest reliability. Others report higher values of CV concerning stride time (e.g., [48], [49]). However, these divergences may be due to rather few stride numbers on short walkways (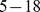 strides). The present study investigates more strides and it has to be emphasized that gait data has to be collected over a reasonable distance to calculate reliability of stride time data [50]. One can speculate that stitched time series are not suitable to calculate CV of stride time.

With respect to nonlinear measures, we found significantly lower fractal scaling exponents in PD patients compared to the control group which fits to the literature [1], [19], [46]. Both fractal scaling exponents 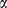 and 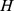 demonstrated equivalent outcomes with respect to the differentiation of PD and CG. Thus, our results underline the sensitivity of fractal methods even if they are based on stitched time series. It has to be emphasised that different polynomial fits were used for both methods (DFA and AFA). The scope of this article was to evaluate the applicability of fractal scaling methods to discriminate PD from CG. Thus, from a methodological point of view, a comparison between the results has to be drawn carefully. To give consideration to this aspect, underlying polynomial fits should be of the same order.

Gait velocity was significantly different between the trials. However, the mean gait speed of the first trial (slowest velocity) was less than 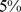 compared to the last trial (fastest velocity). For instance, [42] report their differences between gait speed and the fractal scaling exponent 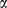 (in the range of 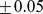) on the basis of 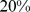 difference from the comfortable self-paced walking speed. We assume that the changes in walking speed may have a marginal effect on the fractal scaling outcome. Furthermore, we found no significant differences on gait velocity between the subject groups. Nevertheless, we recommend that before analysing different gait trials in order to apply the stitching procedure, three practice trials have to be conducted as we have found later trials to reveal more consistent walking speeds. [46] showed that the DFA fractal exponent can be related to age and disease severity. However, we found no linear correlation between UPDRS, a measure of disease severity, and the fractal scaling exponent 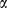. Several reasons may account for this phenomenon. It has been proven that fractal methods are sensitive to identify various and subtle information of the systems behaviour [26], [51]. Furthermore, UPDRS is a sum score of equally weighted items regarding multiple PD specific symptoms. By contrast, gait is a highly complex motor control performance and therefore, a linear interaction between both methods is unlikely. In addition, no significant linear correlation between UPDRS and CV was found which is in contrast to [4]. Our results of having no correlations between the scaling exponent 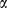 (DFA) and CV support previous findings in PD patients [1] and in older adults [52] which may underline that both parameters (linear versus nonlinear) account for different information in the stride time series. No different scaling regions (linear trend of the log-log-plots in DFA and AFA) were found which is in contrast to [53] who found multifractal scaling for both, PD patients and healthy controls.

The proposed method of stitching together five trials in order to yield longer time series seems to be appropriate in order to distinguish between healthy and pathological gait using nonlinear methods. However, we found higher empirical quantiles and a larger bias, as well as larger mean squared errors concerning surrogate B. One can assume that stitching together the single trials generates a pseudo structure which results in a shift to a positive correlation (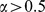). Hence, it can be expected that the presented 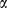-values overestimate the true scaling exponent. This effect was similar in the calculation of 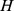. However, our results partly concur with 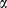-values reported in literature e.g., 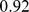 for the control group and 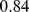 for the PD group [19]. [1] found a fractal scaling exponent of 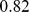 for the PD group which was statistically different from the value of the control group. In general, it was found 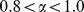 for healthy adults with respect to stride time data [1], [10], [23], which means that our findings are located at the upper end of the reported range.

In accordance with previous studies [30], [31], [54], we found a negative correlation between signal length and 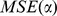 which underlines that the error decreases with increasing signal length. This was not true, however, for surrogate version B. Although stitching together the five trials, signal length was smaller than 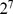 which resulted in large acceptance regions for 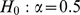. In literature, no consistent recommendations are published concerning the minimum number of strides needed to attain accurate results of gait analysis. [55] propose 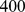 steps [56], suggest 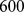 strides, whereas others showed good results of DFA with smaller number of data points [32]. Two methodologically conflicting problems emerge with regard to long time series in gait. First, in clinical standard diagnosis it is hardly possible to measure long distances due to a lack of space and costs required for performing a study [57]. A second problem is the effect of fatigue in patients during prolonged walking. The present study demonstrates a simple procedure which is applicable without these implications. However, from a theoretical point of view, the stitching procedure does not accord to the originally proposed assumption of finding long-range correlations within consecutive strides. The present approach is based on the idea to have measured steady although pathological systems and therefore, sections of the gait process. Therefore, the indicated fractal scaling values are strictly spoken only true for the stitching process. In this study, the true fractal properties of the system (underlying long-term correlations in the signals) were not investigated directly. To account for this aspect, scaling exponents of long continuous recordings of stride time series have to be compared to those scaling values that are obtaind by the stitching procedure. Further research has to be undertaken to elucidate the relationship to long continuous data and to get insight into how many sequences should be measured and how many strides should these sequences consist of.

## Conclusion

The present study demonstrates that applicability of fractal methods in gait analysis is not limited to time series that are collected from prolonged walking conditions. The proposed method enables to create sufficiently long data by stitching together short sequences (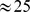 strides) to differentiate between a healthy control group and a group of persons with Parkinsons Disease by use of fractal methods. Hence, this approach is useful, for instance, in the context of clinical investigations that are restricted to short walkways. This work provides a first insight into the agreeability between elaborate gait analysis and clinical suitability. It has to be further elucidated which combination between the number and the length of trials will produce the best results. This systematic analysis would be the next step, to establish fractal analysis as a standardised user-friendly method for clinical standard diagnosis.

## References

[1] Hausdorff JM (2009) Gait dynamics in parkinson's disease: Common and distinct behavior among stride length, gait variability, and fractal-like scaling. CHAOS 19: 026113. 19566273 10.1063/1.3147408 PMC2719464

[2] Hove MJ, Suzuki K, Uchitomi H, Orimo S, Miyake Y (2012) Interactive rythmic auditory stimulation reinstates natural 1/f timing in gait of parkinson's patients. PLOS ONE 7: e32600. 22396783 10.1371/journal.pone.0032600 PMC3292577

[3] Jankovic J, Kapadia AS (2001) Functional decline in parkinson disease. Arch Neurol 58: 1611–1615. 11594919 10.1001/archneur.58.10.1611

[4] Schaafsma JD, Giladi N, Balash Y, Bartels AL, Gurevich T, et al (2003) Gait dynamics in parkinson's disease: relationship to parkinsonian features, falls and response to levodopa. J Neurol Sci 212: 47. 12809998 10.1016/s0022-510x(03)00104-7

[5] Lewis GN, Byblow WD, Walt SE (2000) Stride length regulation in parkinsons disease: the use of extrinsic, visual cues. Brain 123: 2077–2090. 11004125 10.1093/brain/123.10.2077

[6] Morris ME, Mcginley J, Huxham F, Collir J, Iansek R (1999) Constraints on the kinetic, kinematic and spatiotemporal parameters of gait in parkinsons disease. Hum Mov Sci 18: 461–483.

[7] Blin O, Ferrandez AM, Serratrice G (1990) Quantitative analysis of gait in parkinson patients: increased variability of stride length. J Neurol Sci 98: 91–97. 2230833 10.1016/0022-510x(90)90184-o

[8] Hausdorff JM, Cudkowicz ME, Firtion R, Wie JY, Goldberger AL (1998) Gait variability and basal ganglia disorders: stride-to-stride variations of gait cycle timing in parkinsons disease and huntingtons disease. Mov Disord 13: 428–437. 9613733 10.1002/mds.870130310

[9] Hausdorff JM, Mitchell SL, Firtion R, Peng CK, Cudkowicz ME, et al (1997) Altered fractal dynamics of gait: Reduced correlations with aging and huntington's disease. Appl Physiol 82: 262. 10.1152/jappl.1997.82.1.262 9029225

[10] Hausdorff JM, Ashkenazy Y, Peng CK, Ivanov PC, Stanley HE, et al (2001) When human walking becomes random walking: fractal analysis and modeling of gait rhythm fluctuations. Physica A 302: 138–147. 12033228 10.1016/s0378-4371(01)00460-5

[11] Hausdorff JM (2007) Gait dynamics, fractals and falls: finding meaning in the stride-to-stride fluctuations of human walking. Hum Mov Sci 26: 555–589. 17618701 10.1016/j.humov.2007.05.003 PMC2267927

[12] Hollman JH, Childs KB, McNeil ML, Mueller A, Quitter CM, et al (2010) Number of strides required for reliable measurements of pace, rhythm and variability parameters of gait during normal and dual task walking in older individuals. Gait Posture 32: 23–28. 20363136 10.1016/j.gaitpost.2010.02.017

[13] Harbourne R, Stergiou N (2009) Movement varaibility and the use of nonlinear tools: principles to guide physical therapist practice. Phys Ther 89: 267–283. 19168711 10.2522/ptj.20080130 PMC2652347

[14] Schubert P (2013) The application of nonlinear methods to characterize human variability from time series [Schubert P: Die Anwendung nichtlinearer Verfahren zur Charakterisierung der menschlichen Variabilitt aus Zeitreihen]. Dtsch Z Sportmed 64: 132–140.

[15] Herman T, Giladi N, Gurevich T, Hausdorff JM (2005) Gait instability and fractal dynamics of older adults with a ‘cautious’ gait: why do certain older adults walk fearfully? Gait Posture 21: 178–185. 15639397 10.1016/j.gaitpost.2004.01.014

[16] Hausdorff JM (2005) Gait variability: methods, modeling and meaning. J Neuroeng Rehabil 2: 19–27. 16033650 10.1186/1743-0003-2-19 PMC1185560

[17] Chang MD, SEjdic E, Wright V, Chau T (2010) Measures of dynamic stability: Detecting differences between walking overground and on a compliant surface. Hum Mov Sci 29: 977–986. 20655606 10.1016/j.humov.2010.04.009

[18] Hausdorff JM, Purdon PL, Peng CK, Ladin Z, Wei JY, et al (1996) Fractal dynamics of human gait: stability of long-range correlations in stride interval fluctuations. J Appl Physiol 80: 1448. 8727526 10.1152/jappl.1996.80.5.1448

[19] Bartsch R, Plotnik M, Kantelhardt JW, Havlin S, Giladi N, et al (2007) Fluctuation and synchronization of gait intervals and gait force profiles distinguish stages of parkinson's disease. Physica A 383: 455–465. 18163154 10.1016/j.physa.2007.04.120 PMC2156195

[20] Peng CK, Buldyrev SV, Havlin S, Simons M, Stanley HE, et al (1994) Mosaic organization of dna nucleotides. Phys Rev E 49: 1691–1695. 10.1103/physreve.49.1685 9961383

[21] Delignières D, Torre K, Bernard PL (2011) Transition from persistent to anti-persistent correlations in postural sway indicates velocity-based control. Comput Biol 7. 10.1371/journal.pcbi.1001089 PMC3044760 21390333

[22] Peng C, Havlin S, Stanley H, Goldberger A (1995) Quantification of scaling exponents and crossover phenomena in nonstationary hearbeat time series. Chaos 5: 82–87. 11538314 10.1063/1.166141

[23] Dingwell JB, John J, Cusumano JP (2010) Do humans optimally exploit redundancy to control step variability in walking? Plos Comp Biol 6: e1000856. 10.1371/journal.pcbi.1000856 PMC2904769 20657664

[24] Diniz A, Wijnants ML, Torre K, Barreiros J, Crato N, et al (2011) Contemporary theories of 1/f noise in motor control. Hum Mov Sci 30: 889–905. 21196059 10.1016/j.humov.2010.07.006

[25] Goldberger AL, Peng CK, Lipsitz LA (2002) What is physiologic complexity and how does it change with aging and disease? Neurobiol Aging 23: 23–26. 11755014 10.1016/s0197-4580(01)00266-4

[26] Kuznetsov N, Bonnette S, Gao J, Riley MA (2013) Adaptive fractal analysis reveals limits to fractal scaling in center of pressure trajectories. Ann Biomed Eng 41: 1646–1660. 22956160 10.1007/s10439-012-0646-9

[27] Riley MA, Bonnette S, Kuznetsov N, Wallot S, Gao J (2012) A tutorial introduction to adaptive fractal analysis. Front Physiol 3: 371. 23060804 10.3389/fphys.2012.00371 PMC3460370

[28] Gao J, Hu J, Tung WW (2011) Facilitating joint chaos and fractal analysis of biosignals through nonlinear adaptive filtering. PLoS One 6: e24331. 21915312 10.1371/journal.pone.0024331 PMC3167840

[29] Eke A, Hermán P, Bassingthwaighte JB, Raymond GM, Percival D, et al (2000) Physiological time series: distinguishing fractal noises from motions. Pflug Arch Eur J Physiol 439: 403–415. 10.1007/s004249900135 10678736

[30] Eke A, Hermán P, Kocsis L, Kozak LR (2002) Fractal characterization of complexity in temporal physiological signals. Physiol Meas 23: R1–R38. 11876246 10.1088/0967-3334/23/1/201

[31] Kirchner M, Schubert P, Schmidtbleicher D, Haas CT (2012) Evaluation of the temporal structure of postural sway fluctuations based on a comprehensive set of analysis tools. Physica A 391: 4692–4703.

[32] Delignières D, Ramdani S, Lemoine L, Torre K, Fortes M, et al (2006) Fractal analyses for ‘short’ time series: A re-assessment of classical methods. J Math Psychol 50: 525–544.

[33] Beauchet O, Kressig RW, Najafi B, Aminian K, Dubost V, et al (2003) Age-related decline of gait control under a dual-task condition. J Am Geriatr Soc 51: 1187–1188. 10.1046/j.1532-5415.2003.51385.x 12890096

[34] Hollman JH, Kovash FM, Kubic JJ, Linbo RA (2007) Age-realted differences in aspatiotemproal markers of gait stability during dual task walking. Gait Posture 32: 113–119. 10.1016/j.gaitpost.2006.08.005 16959488

[35] Webster KE, Wittwer JE, Feller JA (2005) Validity of the gaitrite® walkway system for the measurement of averaged and individual step parameters of gait. Gait Posture 22: 317–321. 16274913 10.1016/j.gaitpost.2004.10.005

[36] Priest AW, Salamon KB, Hollman JH (2008) Age-related differences in dual task walking: a cross sectional study. J Neuroeng Rehabil 5: 29–37. 19014583 10.1186/1743-0003-5-29 PMC2607296

[37] Chen Z, Ivanov P, Hu K, Stanley HE (2002) Effect of nonstationarities on detrended fluctuation analysis. Physical Rev E 65: 041107-1 to 041107-15. 10.1103/PhysRevE.65.041107 12005806

[38] Kressig RW, Beauchet O (2006) Guidelines for clinical applications of spatio-temporal gait analysis in older adults. Aging Clin Exp Res 18: 174–176. 16702791 10.1007/BF03327437

[39] Lindemann U, Najafi B, Jijlstra W, Hauer K, Muche R, et al (2008) Distance to achieve steady state walking speed in frail elderly persons. Gait Posture 27: 91–96. 17383185 10.1016/j.gaitpost.2007.02.005

[40] Bloem BR, Bhatia KP (2004) Gait and balance in basal ganglia disorders. In: Bronstein A, Brandt T, Woollacott MH, Nutt J, editors, Clinical Disorders of Balance, Posture and Gait, Arnold: London. pp. 173–206.

[41] Theiler J, Eubank S, Longtin A, Galdrikian B, Farmer JD (1992) Testing for nonlinearity in time series: the method of surrogate data. Physica D 58: 77–94.

[42] Jordan K, Challis JH, Newell KM (2007) Walking speed influences on gait cycle variability. Gait Posture 26: 128–134. 16982195 10.1016/j.gaitpost.2006.08.010

[43] Winter DA (1993) Biomechanical motor patterns in normal walking. J Mot Behav 15: 302330. 10.1080/00222895.1983.10735302 15151864

[44] Frenkel-Toledo S, Giladi N, Peretz C, Herman T, Gruendlinger L, et al (2005) Effect of gait speed on gait rhythmicity in parkinson's disease: variability of stride time and swing time respond differently. J Neuroeng Rehabil 2: 23. 16053531 10.1186/1743-0003-2-23 PMC1188069

[45] Hausdorff JM, Lertratanakul A, Cudkowicz ME, Peterson AL, Kaliton D, et al (2000) Dynamic markers of altered gait rhythm in amyotrophic lateral sclerosis. J Appl Physiol 88: 2045–2053. 10846017 10.1152/jappl.2000.88.6.2045

[46] Ota L, Uchitomi H, Suzuki K, Miyake Y, Hove MJ, et al.. (2012) Evaluation of severity of parkinson's disease using stride interval variability. In: International Conference on Complex Medical Engineering. pp. 521–526.

[47] Beauchet O, Freiberger E, Annweiler C, Kressig RW, Herrmann FR, et al (2011) Test-retest reliability of stride time variability while dual tasking in healthy and demented adults with frontotemporal degeneration. J Neuroeng Rehabil 8: 37–42. 21745370 10.1186/1743-0003-8-37 PMC3156726

[48] Brach JS, Perera S, Studenski S, Newman AB (2008) Test-retest reliability of stride time variability while dual tasking in healthy and demented adults with frontotemporal degeneration. Arch Phys Med Rehabil 89: 2293–2296.19061741

[49] Montero-Odasso M, Casas A, Hansen KT, Bilski P, Gutmanis I, et al (2009) Quantitative gait analysis under dual-task in older people with mild cognitive impairment: a reliability study. J Neuroeng Rehabil 21: 35. 10.1186/1743-0003-6-35 PMC2754991 19772593

[50] Lord S, Howe T, Greenland J, Simpson L, Rochester L (2011) Gait variability in older adults: a structured review of testing protocol and clinimetric properties. Gait Posture 34: 443–450. 21920755 10.1016/j.gaitpost.2011.07.010

[51] Goldberger AL, Amaral LA, Hausdorff JM, Ivanov P, Peng CK, et al (2002) Fractal dynamics in physiology: alterations with disease and aging. Proc Natl Acad Sci U S A 99 Suppl 1: 2466–2472. 11875196 10.1073/pnas.012579499 PMC128562

[52] Cavanaugh (2010) Nonlinear analysis of ambulatory activity patterns in community-dwelling older adults. J Gerontol A Biol Sci Med Sci 65: 197–203. 19822625 10.1093/gerona/glp144 PMC2806237

[53] Dutta S, Ghosh D, Chatterjee S (2013) Multifractal detrended fluctuation analysis of human gait diseases. Front Physiol 4: 274. 24109454 10.3389/fphys.2013.00274 PMC3791390

[54] Weron R (2002) Estimating long-range dependence: finite sample properties and confidence intervals. Physica A 312: 285–299.

[55] Owings TM, Grabiner MD (2003) Measuring step kinematic variability on an instrumented treadmill: how many steps are enough? J Biomech 36: 1215–1218. 12831749 10.1016/s0021-9290(03)00108-8

[56] Damouras S, Chang MD, Sejdic E, Chau T (2010) An empirical examination of detrended fluctuation analysis for gait data. Gait Posture 31: 336–340. 20060298 10.1016/j.gaitpost.2009.12.002

[57] Simon SR (2004) Quantification of human motion: gait analysis-benefits and limitations to its application to clinical problems. J Biomech 37: 1869–1880. 15519595 10.1016/j.jbiomech.2004.02.047

